# Plasma lipidomics of tuberculosis patients: altered phosphatidylcholine remodeling

**DOI:** 10.4155/fsoa-2017-0011

**Published:** 2017-10-20

**Authors:** Paul L Wood, Soumya Tippireddy, Joshua Feriante

**Affiliations:** 1 Metabolomics Unit, Department of Physiology & Pharmacology, DeBusk College of Osteopathic Medicine, Lincoln Memorial University, 6965 Cumberland Gap Parkway, Harrogate, TN 37752, USA; 2 College of Veterinary Medicine, Lincoln Memorial University, 6965 Cumberland Gap Parkway, Harrogate, TN 37752, USA

**Keywords:** lipid remodeling, lysophosphatidylcholines, phosphatidylcholines, phosphatidylglycerols, plasma, tuberculosis

## Abstract

**Aim:**

Decreased circulating levels of lysophosphatidylcholines have been monitored in the serum of tuberculosis (TB) patients. However, the etiology of these findings has not been explored and other critical lung surfactant lipids have not been examined.

**Materials & methods:**

We undertook a lipidomics analysis of 30 controls and 30 TB patients, utilizing a high-resolution mass spectrometric analytical platform that assays over 1800 lipids.

**Findings:**

As previously reported, we found decrements in the plasma levels of lysophosphatidylcholines in TB patients. In addition, we report for the first time that there are increases in the plasma levels of phosphatidylcholines and phosphatidylglycerols in TB patients.

**Conclusion:**

These data suggest that TB results in altered glycerophosphocholine remodeling involving deacylation–reacylation reactions at sn-2 of the glycerol backbone. Such alterations in lipid remodeling have the potential to exert negative effects on the function of lung surfactant, on signal transduction mechanisms and membrane structural lipid architecture in TB patients.

## Lay abstract

On investigating tuberculosis patients, we found altered metabolism of structural lipids that are essential to lung function. These new biomarkers may be useful to monitor the efficacy of current and new patient treatment strategies in the future.

*Mycobacterium tuberculosis* parasitizes host cells and exploits host nutrients to sustain metabolism and for the biosynthesis of bacterial structural biomolecules, including membrane lipids [1,2]. In this regard two previous studies have demonstrated decrements in the levels of lysophosphatidylcholines in tuberculosis (TB) host plasma [3,4]. These data are suggesting that TB can alter glycerophospholipid remodeling involving deacylation–reacylation reactions at sn-2 of the glycerol backbone [5–7]. However, the structural phosphatidylcholines that are metabolized to lysophosphatidylcholines have not been characterized in the plasma of TB patients. Therefore, we undertook a nontargeted lipidomics evaluation of plasma from TB patients utilizing a high-resolution (<3 p.p.m. mass error) mass spectrometric analytical platform to evaluate over 1800 individual lipids across a number of lipid subclasses [7,8]. This approach allowed us to dissect the mechanism involved in the altered levels of lysophosphatidylcholines and to examine specific phosphatidylcholines and phosphatidylglycerols that are also essential components of lung surfactant.

## Materials & methods

### Clinical samples

The plasma samples were obtained from the WHO Tuberculosis Specimen Bank via FIND [9], which manages this biorepository. This included 30 controls and 30 African TB patients. Patient demographics are presented in Table 1.

**Table 1.** **Patient demographics of control and tuberculosis subjects.**

**Parameter**	**Control group**	**Active TB group**
Age (years) [range]	38.9 ± 3.1 [25–81]	47.5 ± 2.6 [25–74]

Gender	13 F/17 M	15 F/15 M

Mildly Ill		73%

Moderately Ill		27%

Cough		100%

Fever		56%

Chest pain		78%

Dyspnea		71%

### Lipidomics analyses of plasma

After thawing,100 μl aliquots of plasma were mixed with 1 ml of methanol containing stable isotope internal standards, followed by 1 ml of water and 2 ml of methyl tert-butyl ether [7,8]. The tubes were vigorously shaken at room temperature for 30 min prior to centrifugation at 3000 × *g* for 10 min. One milliliter of the upper organic layer was dried by centrifugal vacuum evaporation prior to dissolution in 150 μl of isopropanol:methanol:chloroform (4:2:1) containing 15 mM ammonium acetate. Shotgun ESI lipidomics (5 μl/min) was performed utilizing high-resolution data acquisition (140,000 at 200 amu; 0.3–3 p.p.m. mass error; *m/z* 200–1400) with an orbitrap mass spectrometer (Thermo Q Exactive).

In negative ion ESI (3.2 kV, capillary temperature of 320°C, sheath gas of 10), the anions of phosphatidylethanolamines, lysophosphatidylethanolamines, ethanolamine plasmalogens, phosphatidylglycerols, phosphatidylinositols, phosphatidylserines, sterol sulfates, free fatty acids, perfluoroalkyl toxins, cyclic phosphatidic acids and the [M + HCOO]^-^ anions of ceramides were monitored. In positive ion ESI (4.3 kV, capillary temperature of 320°C, sheath gas of 10), the cations of choline plasmalogens (PlsCh), phosphatidylcholines, lysophosphatidylcholines, ceramides and sphingomyelins and the ammonium adducts of diacylglycerols and triacylglycerols were monitored.

Data are presented as R-values (ratio of the endogenous lipid peak area to the peak area of an appropriate internal standard) per 100 μl of plasma (n = 30 per group).

### Statistics

Data were analyzed utilizing the two-tailed *t*-test.

## Results

### Phosphatidylcholines & lysophosphatidylcholines

**Figure 1.**
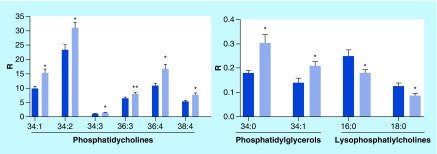
**Augmented levels of phosphatidylcholines and phosphatidylglycerols with parallel decreases in lysophosphatidylcholines in the plasma of tuberculosis patients.** R: Ratio of peak area of endogenous lipid/peak area of stable isotope internal standard per 0.1 ml of plasma (mean ± SEM; n = 30 per group). Nomenclature example: 34:1 = 34 carbons and 1 double bond. *p < 0.01 versus controls; **p < 0.05 versus controls. SEM: Standard error of the mean.

**Figure 2.**
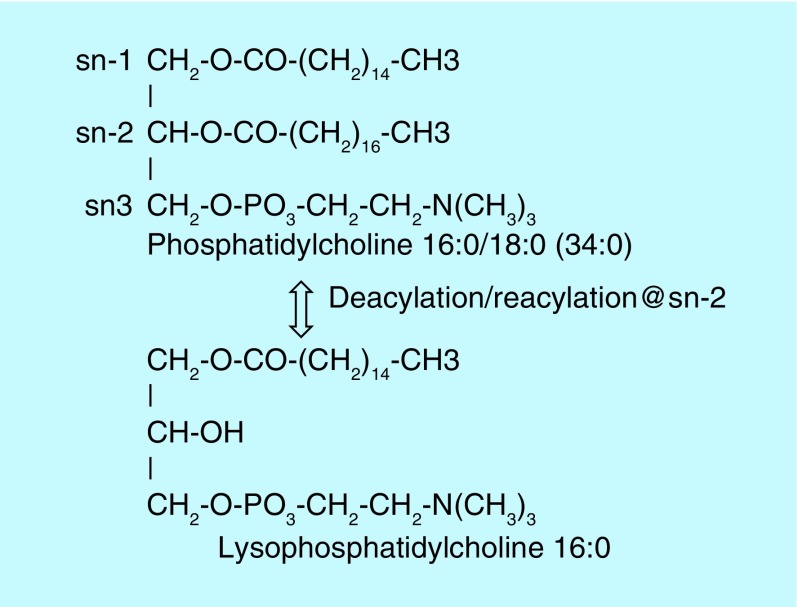
**Schematic of glycerophosphocholine remodeling via deacylation–reacylation reactions at sn-2 of the glycerol backbone to generate lysophosphatidylcholines.** Lipid remodeling predominates at sn-2 in normal conditions and involves tight coupling of the deacylation and reacylation reactions.

Elevated plasma levels of a number of phosphatidylcholines were found in the plasma of TB patients (Figure 1). This included phosphatidylcholines with mono-, di-, tri- and tetra-unsaturated fatty acid substitutions but not polyunsaturated fatty acids. Increases in phosphatidylcholines were paralleled by decrements in lysophosphatidylcholines, the deacylated metabolites of phosphatidylcholines (Figures 1 & 2).

### Phosphatidylglycerols

Levels of phosphatidylglycerol 34:0 and 34:1 were also elevated in the plasma of TB patients (Figure 1).

### Other lipids

A large number of monitored lipids were unaltered in TB patient plasma samples. These included free fatty acids, very-long-chain fatty acids, dicarboxylic acids, ceramides, phosphatidic acids, lysophosphatidic acids, cyclic phosphatidic acids, choline and ethanolamine plasmalogens, phosphatidylethanolamines, lysophosphatidylethanolamines, phosphatidylinositols, diacylglycerols, triacylglycerols, acetylcarnitines and sphingomyelins.

## Discussion

Human phosphatidylcholines possess mainly palmitic (16:0), stearic (18:0) or oleic acid (18:1) at sn-1, while the fatty acid substitution at sn-2 is much more varied, with unsaturated and polyunsaturated fatty acid substituents predominating. Lipid remodeling at sn-2 of phosphatidylcholines is a dynamic process, which is responsible for the generation of a large family of phosphatidylcholines [6–8,10–11]. This involves removal of sn-2 fatty acids by 2-acyl hydrolases, phospholipase A2 (EC 3.1.1.4) and acylglycerol lipase (EC 3.1.1.23), prior to reacylation, with alternate fatty acids, by acyl-CoA:lysophospholipid 2-acyltransferases [12,13]. Evaluations of alterations in phospholipase A2 in TB patients are complicated since there are over 26 phospholipase A2 genes and multiple enzyme isoforms [14]. However, the specificity of the alterations we observed indicates that this is a research problem worthy of further study. The changes in lipid metabolism were specific to phosphatidylcholines and phosphatidylglycerols, with no changes in the levels of phosphatidylserines, phosphatidylethanolamines or phosphatidylinositols. Similarly, levels of choline and ethanolamine plasmalogens were unchanged, indicating that lipid remodeling of these membrane lipids is unaltered, and further demonstrates the specificity of the observed lipid changes.

With regard to other lung infections, lysophosphatidylcholines are increased and lysophosphatidylethanolamines are decreased in the plasma of pneumonia patients [15], while studies of influenza virus have demonstrated a unique alteration in host plasmalogen metabolism [16]. These findings further validate the specificity of our findings. Similarly, these studies did not report alterations in phosphatidylglycerol metabolism, while we noted augmented levels of these lipids that are critical components of lung surfactant [17].

## Conclusion

In summary, our data suggest that an uncoupling of phosphatidylcholine-coordinated deacylation–reacylation reactions occurs in TB patients resulting in altered phosphatidylcholine and phosphatidylglycerol metabolism. This uncoupling can have diverse metabolic consequences including altered signal transduction cascades and altered membrane composition in phosphatidylcholine and phosphatidylglycerol content. These alterations in membrane ultrastructure could lead to altered lung surfactant properties as well as changes in enzyme, transporter and ion channel function in membranes [5,6]. The relative roles of these metabolic defects in patient recovery remain to be determined.

## Future perspective

Our findings offer the potential for utilizing new biomarkers to monitor the efficacy of current and new patient treatment strategies in the future. Our data also suggest that new therapeutic strategies related to alterations in glycerophospholipid metabolism may be worthy of investigation.

**Executive summary**

- Decreased circulating levels of lysophosphatidylcholines were monitored in the plasma of tuberculosis (TB) patients.
- Increased circulating levels of phosphatidylcholines and phosphatidylglycerols were measured in the plasma of TB patients.
- These data suggest that an uncoupling of phosphatidylcholine-coordinated deacylation–reacylation reactions occurs in TB patients.
- Our findings offer the potential for utilizing new biomarkers to monitor the efficacy of current and new patient treatment strategies in the future.
